# The use and effect of virtual reality as a non-pharmacological intervention for behavioural and psychological symptoms of dementia: a systematic review and meta-analysis

**DOI:** 10.1093/ageing/afaf117

**Published:** 2025-05-12

**Authors:** Li-Chin Wang, Amy Montgomery, Peter Smerdely, Olivia Paulik, Cherie Barton, Elizabeth Halcomb, Heidi Hoi Ying Hui, Carolyn Pieri, Maria Rios Lopez, Judeil Teus, Gemma McErlean

**Affiliations:** Department of Aged Care, Gosford Hospital, Gosford, Australia; School of Nursing, Faculty of Science, Medicine and Health, University of Wollongong, Wollongong, Australia; Department of Aged Care, St. George Hospital, Kogarah, Australia; Department of Aged Care, St. George Hospital, Kogarah, Australia; Department of Aged Care, St. George Hospital, Kogarah, Australia; School of Nursing, Faculty of Science, Medicine and Health, University of Wollongong, Wollongong, Australia; Center for Research in Nursing and Health, St. George Hospital, Kogarah, Australia; Center for Research in Nursing and Health, St. George Hospital, Kogarah, Australia; Department of Aged Care, St. George Hospital, Kogarah, Australia; School of Nursing, Faculty of Science, Medicine and Health, University of Wollongong, Wollongong, Australia; Center for Research in Nursing and Health, St. George Hospital, Kogarah, Australia; School of Nursing, Faculty of Science, Medicine and Health, University of Wollongong, Wollongong, Australia; Center for Research in Nursing and Health, St. George Hospital, Kogarah, Australia

**Keywords:** virtual reality, behavioural and psychological symptoms of dementia, non-pharmacological, systematic review, older people

## Abstract

**Background:**

Behavioural and psychological symptoms of dementia (BPSD) are complex neuropsychiatric symptoms that contribute to caregiver strain, increased rates of institutionalisation and reduced quality of life. Virtual reality (VR) has gained interest as a non-pharmacological approach to potentially reduce BPSD severity.

**Objective:**

This review sought to synthesise evidence on the effectiveness of VR in reducing BPSD severity, while exploring its acceptability, safety, and optimal dosage in dementia care.

**Methods:**

MEDLINE, EMBASE, CINAHL and SCOPUS were searched for randomised and quasi-experimental trials assessing VR’s effect on BPSD. JBI critical appraisal checklists were used to assess methodological quality. Findings were presented narratively, with meta-analysis performed on a subset of BPSD symptoms where data were available.

**Results:**

Of the ten included studies, four found no significant change in overall BPSD. Mixed findings were observed for individual BPSD symptoms. Meta-analysis showed a significant reduction in depressive symptoms (mean diff −0.38, *P*= .026) and no reduction in agitation (mean diff 1.87, *P* = .2). Two studies reported reduced aggression and mixed findings were found for anxiety. Reduced apathy was observed in one study following each VR session and during the session in another. VR was generally well-accepted with few side effects reported.

**Conclusion:**

VR appears to be an acceptable non-pharmacological intervention for BPSD reduction. However, the limited available studies, methodological variations and quality issues suggest the need for future larger-scale research to confirm its efficacy and effectiveness.

## Key points

- Virtual reality may reduce the severity of behavioural and psychological symptoms of dementia, particularly in reducing aggression, depressive symptoms, and apathy.
- Favourable acceptability and minimal side effects were observed across various clinical settings.
- The findings are constrained by the limited number of studies available, varied methodology and quality limitations, highlighting the need for future robust research.

## Introduction

Dementia is a complex syndrome marked by a progressive decline in the cognitive domains required for independent functioning [1]. The term behavioural and psychological symptoms of dementia (BPSD) is used to describe the range of non-cognitive symptoms commonly experienced by people living with dementia. BPSD is a widely recognised term in clinical practice, dementia care guidelines, and health policies [2, 3]. BPSD encompasses various neuropsychiatric symptoms, including agitation, apathy, psychosis, aggression, and sexual disinhibition [4]. Up to 80% of people living with dementia may develop BPSD over their disease course [5]. These symptoms can lead to physical injuries, psychological distress and increased caregiver burden, leading to higher rates of institutionalisation and poorer quality of life [6].

While antipsychotic medications are often used to manage BPSD in clinical practice, they have limited efficacy and potentially serious side effects [7]. A recent Cochrane review found weak evidence for the use of antipsychotics in reducing agitation and psychosis in dementia, with observed effectiveness likely reflecting natural symptom improvement as observed in the placebo group [8]. Due to the risks of somnolence, extrapyramidal features and even death associated with antipsychotics [8], there is growing emphasis on exploring non-pharmacological approaches to reduce BPSD.

Non-pharmacological therapies are the preferred first-line treatment for BPSD in most dementia management guidelines [9]. Interventions ranging from traditional approaches with behavioural therapy, caregiver training and psychosocial interventions to newer therapies incorporating art, music and technology all show promising results [10]. However, despite several meta-analyses, there is no consensus on the most effective non-pharmacological approach to managing BPSD [11, 12]. Barriers to broader implementation of non-pharmacological therapies persist due to funding constraints, staffing challenges and reluctance from family or staff [13]. Therefore, exploring non-pharmacological interventions that are easy to implement, resource-efficient, and effective in reducing BPSD is crucial.

Over the past decade, virtual reality (VR) technologies have emerged as a novel therapy for dementia. VR is a computer-generated three-dimensional (3D) simulation that allows users to experience alternate physical spaces [14]. Fully immersive VR is accessed through computers with 3D screen displays or head-mounted displays (HMD) with headphones and movement sensors [15]. The virtual environment updates in real time to reflect the user’s actions [16]. This immersive experience encourages high engagement with new experiences and social interactions [17], which can otherwise be challenging to achieve for those with dementia due to impaired communication skills.

The clinical potential of VR in dementia is broad, encompassing enhanced mobility, falls prevention, cognitive training [18], and early identification of cognitive decline [19]. The feasibility and acceptability of older people with frailty wearing HMD have been shown regardless of mobility or cognitive abilities [20]. A recent systematic review [21] demonstrated that VR improved cognition, quality of life and activities of daily living for older people with dementia. However, there is limited literature on the effect of VR on BPSD, and no systematic review has assessed VR as a non-pharmacological therapy for reducing BPSD severity. Therefore, this systematic review aimed to evaluate the effectiveness of VR in reducing BPSD severity, as well as exploring its acceptability, safety and optimal dosage.

## Methods

This systematic review was reported according to the Preferred Reporting Items for Systematic Review and Meta-analysis (PRISMA) statement [22]. The protocol was registered in the International Prospective Register of Systematic Reviews database (CRD42024523848). Changes to the registered protocol include an additional focus on secondary outcomes of acceptability and adverse effects of VR.

### Search strategy

A search strategy was developed for MEDLINE and CINAHL, which was then adapted for MEDLINE, EMBASE, and SCOPUS (see Appendix 1 in the Supplementary Data section for the full search strategy). Search terms included VR, dementia, BPSD. Other keywords describing BPSD, such as challenging behaviour, agitation, apathy and disinhibition, were also used. Reference lists of related sources were screened to identify additional relevant studies.

### Inclusion criteria

Only studies published in the English language between 2014 and 2024 were included due to the rapid evolution of VR technology. The inclusion criteria were (i) Participants: studies which included people with dementia of any stage and aetiology who experience BPSD; (ii) Intervention: VR interventions including fully, semi- and non-immersive forms; (iii) Comparator: studies with or without control groups were included; (iv) Outcome: any measure of change in BPSD from baseline following intervention; (v) Study types: experimental studies including RCTs and quasi-experimental studies. These inclusion criteria were kept purposely broad, given the limited literature and relative infancy of research in this area, to capture a breadth of research about the intervention of interest in this population group.

### Study selection

All retrieved papers were managed using Covidence software [23]. After removing duplicates, two independent reviewers (LW and AM) screened the titles, abstracts and full texts of selected articles based on the inclusion criteria. Any disagreements between reviewers at any stage were addressed through discussion or referred to a third reviewer for resolution through consensus (GM).

### Quality assessment

The methodological quality of the included studies was assessed using the JBI critical appraisal checklists for RCTs [24] and quasi-experimental studies [25] by two independent reviewers (LW and AM). The checklist consisted of thirteen and nine questions, respectively, with four possible answers (yes, no, unclear and not applicable). These questions assessed the quality, validity and applicability of the studies. Disagreements between the two reviewers (LW and AM) were resolved by discussing or involving a third reviewer (GM).

### Data extraction

Data items were extracted from full-text articles using a modified extraction template on Covidence [23] and included participant characteristics, details of the interventions, acceptability, any adverse events, as well as significant outcomes (Table 1). One reviewer (LW) extracted and entered the data into the template form, while the other (AM) cross-checked the data. Any errors were corrected through consensus.

**Table 1:** Characteristics of included studies.

Citation (Country)	Design and setting	Participants	Intervention/Control	Data collection	Acceptability	Adverse events	Key outcomes
Appel et al., 2024 [26] (Canada)	RCT Acute hospital, medical ward.	Sixty-nine participants aged ≥65 years with dementia. Cognitive impairment ranged from mild to severe. Intervention group (*n* = 34), 64.7% female. Control group (*n* = 35), 65.7% female. Baseline neuropsychiatric inventory (NPI) score not reported.	Individual fully immersive VR sessions with HMD. Each session lasted up to 20 mins with short 360-degree films including natural elements, motion, and sound. Participants and caregivers could collaboratively select the scene. Standard care based on hospital guidelines.	Key terms relating to BPSD symptoms from daily nursing notes were categorised into three predetermined clusters (NPI-10 like, aggression and wandering). Outcomes were measured as the difference in mean number of events between ‘before’ the first VR session and ‘after’ the last VR session/discharge.	Intervention group only: 85% (*n* = 29) completed ≥1 session. Mean 1.6 sessions Average session length 6.8 mins. 15% (*n* = 5) refused and completed no VR sessions.	Two adverse events (stroke and death) unrelated to VR therapy in intervention group. Participants reported feeling ‘a bit’ nauseous in 2% (*n* = 1/47) of the VR sessions conducted.	Intervention group had significant reduction in the aggression cluster of BPSDs (*P* = .01), compared to the control group. No significant difference between the groups when comparing the NPI-10 like cluster (*P* = .28) and wandering cluster (*P* = .7).
Appel et al., 2021 [20] (Canada)	Quasi-experimental Acute hospital, medical ward.	Ten participants aged >65 years, with dementia. Mean age 86.5 years, 80% female. Dementia severity ranged from mild to severe. Mean NPI score (12 item) rated by caregiver based on behaviours for the four to six weeks prior to intervention was 13 (SD 8.87).	Individual fully immersive VR sessions with HMD. Sequence of five short 360-degree video clips (one to three mins each) for ≤20 min each session. Various natural scenes (rocky lakeshore, sunny forest, floating icebergs, and sunny beaches). Participants could loop through the film sequence.	BPSD behaviours were measured by count from nursing notes and categorised into 14 predetermined categories (e.g. agitation, refusing or declining care, violence, wandering and vocalisations). Mood assessed pre and immediately post-sessions using a survey consisting of ‘yes’ or ‘no’ responses to eight moods (e.g. calm, sad, energetic, lonely or worried). Average length of stay was 11.1 days (SD 7.2).	Eighteen VR sessions conducted. Seventy percent opted for additional VR sessions after the first session. Mean duration 6.2 min (SD 5.5). No participants kept the HMD on for 20 min. Sixty-seven percent (*n* = 12) chose to remove the VR headset without distress, and 17% (*n* = 3) removed headsets due to low interest.	One (10%) participant experienced self-limiting dizziness with mild nausea. One (10%) participant found the headset too heavy.	BPSD behaviours displayed during hospitalisation included agitation in 80% (*n* = 8), insomnia (80%, *n* = 8), vocalisations (70%, *n* = 7), wandering (60%, *n* = 6), refusal of medical care (60%, *n* = 6), mood symptoms of depression or anxiety (30%, *n* = 3), violent behaviour (20%, *n* = 2) and perceptual disturbances (10%, *n* = 1). No pre-post-intervention comparison or analysis was conducted. Mood post-VR was calm in 83% (*n* = 15) sessions, sad/upset in 17% (*n* = 3) sessions, worried in 17% (*n* = 3) sessions, and tired in 22% (*n* = 4) sessions. 60% (*n* = 6) participants had difficulty answering the survey relying on caregiver input or body language.
Brimelow et al., 2022 [27] (Australia)	Quasi-experimental Residential aged care facility (RACF).	Twenty-five participants of varying cognitive capacity and dementia. Mean age not reported. 60% (*n* = 15) were female. 32% (*n* = 8) had an existing anxiety or depression diagnosis. Control group had comparable cognitive impairment, age and gender in the same RACF.	Group sessions of five participants conducted twice weekly for three weeks (total six sessions) using fully immersive VR with HMD. Each session lasted 60 min (approximately 10 min of VR experience). VR scenes included beach, rainforest, or the typical London household. Residents could choose scenes and given more options in subsequent weeks. Limited control group (*n* = 7) ran for two sessions using static pictures.	Person environmental apathy rating (PEAR) assessed apathy before and after the intervention. CSDD, GAD-7 and CMAI. OERS and visual analogue scale (VAS) assessed mood before and immediately after each session.	Sixty-four percent (*n* = 16) participants completed 4–6 VR sessions due to personal preference and fluctuating health.	Two participants (8%) reported mild adverse events including headache and giddiness. VR headset caused discomfort in two participants (8%) due to ill fit.	In the intervention group: There was reduction in apathy overall (*P* < .001), at sessions 1 (*P* = .001), 3 (*P* = .031) and 6 (*P* = .041). There was a reduction in mean depression scores (*P* = .009). There was no significant change in anxiety (*P* = .681). There was an increase in agitation (*P* = .003), with sub-analysis showing an increase in repetitive sentences (*P* = .001), complaining (*P* = .001) and negativism (*P* = .001). There was no difference in behavioural incident frequency (p = 0.003). There were improved mean mood scores (*P* < .001) and pleasure overall in OERS (*P* < .001). In the limited control group: There were no changes in apathy at session one (*P* = .114) or two (*P* = .593). There were no changes in pleasure at session one (*P* = .098) or two (*P* = .593).
Clay et al., 2023 [28] (UK)	Quasi-experimental Dementia care unit, (average length of stay of 6–12 weeks).	Fourteen participants with dementia. Mean age 76.8 and 57% (*n* = 8) were female. Mean estimated Clinical Dementia Rating (CDR) scale 2.4 (0–3). Primary reason for admission was aggression in 84% (*n* = 16).	Individual sessions offered as activities within routine OT sensory sessions over 13 weeks. Fully immersive VR with HMD. Three-minute videos of nature scenes from around the world including beaches, waterfalls and forest scenes. Scenes were either chosen for participants or self-chosen.	Clinical records were reviewed retrospectively using the NPI as framework for estimating neuropsychiatric symptoms across 9 domains. Neuropsychiatric symptoms were examined for the period 24 h before and after each VR session.	Nine (64%) participants completed multiple sessions, with the first session 30–60 s long and second session 2–5 min long. 25 sessions completed.	One (7%) participant, with multiple previous falls, fell without injury >12 h after the session.	There was no difference in BPSD in each of the following NPI domains: delusions (*P* = .57), hallucinations (*P* = 1.00), aggression/irritability (*P* = .57), depression (*P* = .57), anxiety (*P* = .71), elation (*P* = 1.00), apathy (*P* = .99), aberrant motor behaviour (*P* = .10) and sleep (*P* = .42).
Coelho et al., 2020 [29] (Portugal)	Quasi-experimental Community setting (local health and social service provider).	Nine participants aged >65 years with a clinical diagnosis of dementia. Mean age 85.6 and 66.7% (*n* = 6) were female. Global Deterioration Scale (GDS): stage four 33.3% (*n* = 3), stage five 33.3% (*n* = 3), stage six 33.3% (*n* = 3). Mean Montreal Cognitive Assessment (MOCA) was 7.2 out of 30 (SD 5.3).	Four individual reminiscence sessions conducted over two weeks using fully immersive VR with headset. Each session lasted 10 min. Interviews (30–60 min) conducted with participants and family members prior to VR sessions to identify possible video recording venues that could elicit positive memories. Locations selected for filming included specific streets, gardens, churches, or historical landmarks of individual relevance. GoPro Fusion 360 camera used to film the selected locations for 20–30 min each.	NPI scores were used to assess BPSD pre- and immediately post each VR intervention. BPSD during the sessions were assessed by a pre-prepared scale created by the researchers that incorporated CSDD and NPI with observed symptoms such as sad expressions or hand wringing rated as absent, intermittent, moderate or severe.	Seventy-eight percent (*n* = 7) took part in all four sessions. One (11%) participant missed two sessions due to health issues and one (11%) participant missed the fourth session due to reporting the previous sessions unpleasant.	Two (22%) participants reported eye strain and head fullness. One (11%) participant reported blurred vision. Severe simulator sickness was not reported.	Mean NPI score was 9.2 (SD 5.3) pre VR and 9.7 (SD 5.3) post VR. No significant difference in BPSD pre and post-VR (*P* = .9). During the VR intervention, agitation was observed in 33% (*n* = 3) and anxiety in 22.2% (*n* = 3) of participants in session one. In session four, 28.6 (*n* = 2) participants experienced anxiety and agitation.
Huang et al., 2022 [30] (Taiwan)	Quasi-experimental Non-hospital based dementia care unit	Twenty participants with dementia of any cause based on National Institute on Aging—Alzheimer’s Association (NIA-AA) guidelines. Mean age 79 and 55% (*n* = 11) were female. Dementia severity according to CDR ranged from very mild (10%, *n* = 2), mild 75%, *n* = 15) to moderate (15%, *n* = 3). The mean Mini-Mental State Examination (MMSE) score was 15.4 out of 30 (SD 5.5)	Individual reminiscence-based sessions administered twice per week over three months using fully immersive VR with headset. Each session lasted 12–14 min. VR content created based upon photographs and narration provided by caregivers and created based on a historical type of residence commonly found in Taiwan. Participants were able to hold rice to feed chickens with a controller.	Centre for Epidemiological Studies Depression (CESD) scale was used to assess depressive symptoms pre-and immediately post each VR intervention.	Not reported.	Not reported.	Reduced depressive symptoms following VR (*P* = .008), with mean CESD of 6.15 pre-VR and 3.15 post-VR.
Matsangidou et al., 2023 [31] (Cyprus)	Quasi-experimental Dementia-specific RACF	Twenty participants with dementia diagnosis. Mean age 73.15 years and 65% (*n* = 13) were female. The mean MMSE score was 15.1 out of 30 (SD 6.16).	Individual session using fully immersive VR with headset. Each session lasted up to 15 min. Fourteen virtual environments such as nature scenes, travel destinations and cooking shows.	The following were collected before and immediately after each VR session: Overt Aggression Scale—Modified for Neurorehabilitation (OAS-MNR) was used to assess aggression, OERS and VAS.	Eighty-five (*n* = 17) participants completed the 15 min VR session and 85% (*n* = 17) requested longer exposure times. The mean exposure time was 14.38 min.	Not reported	Reduced aggression frequency and severity during and after VR exposure (no *P*-value was reported; aggregate aggression score from nine before VR to zero during and after VR). Increased pleasure from before to after VR (*P* = .002). Reduced anger from before to after VR (*P* < .01). Reduced anxiety and fear from before to after VR (*P* = .001). Decrease in negative emotions and increase in positive emotions from before to after VR (*P* < .001).
Moyle et al., 2018 [32] (Australia)	Quasi-experimental RACF	Ten participants aged >60 years with a documented diagnosis of dementia. Mean age 89 years and 70% (*n* = 7) were female. Mean Psychogeriatric Assessment Scale (PAS) score of 13. Mean duration in facility 21.5 months.	Individual VR session. Exposure for a maximum of 15 min. Exposure to VR forest experience.	Data was collected before and immediately after one VR session. PEAR scale was used to assess apathy. OERS score was used to assess the five emotions (Pleasure, alertness, anger, anxiety/fear and sadness).	Mean time spent interacting with VR forest was 10.2 min.	Not reported.	No difference in apathy between pre and post-VR (*P* > .05). There was reduced apathy during VR compared to before the intervention (*P* = .01). During the VR experience, there was more pleasure (*P* = .008) and more alertness (*P* < .001) than OERS scores previously established for people with dementia in an activity context [33].
Sanchez-Nieto et al., 2023 [34](Spain)	Quasi-experimental Community setting (Day Stay Unit)	Three participants with diagnosis of dementia and GDS between five and six. Mean age 79.3 years and 100% (*n* = 3) were female.	Individual sessions administered three days per week for three weeks using fully immersive VR with headset. Exposed to VR for 15 min. Colour themed natural environments including fall, meadows and ocean.	Data was collected pre- and 5-weeks post-intervention. NPI-questionnaire (NPI-Q) was used to assess BPSD symptoms. Hamilton Anxiety Rating Scale (HARS) was used to assess anxiety. State–Trait Anxiety Inventory reduced version (STAIr) was used to assess anxiety pre and immediately after each session.	All three participants (100%) completed their VR sessions as planned.	Not reported.	No difference in neuropsychiatric symptoms pre and post-intervention (*P* = 0.68). No difference in psychological (*P* = 0.087) and somatic anxiety (*P* = 0.5) pre and post-intervention based on HARS. No difference in state of anxiety pre- and post-based on STAIr (*P* = 0.73).
Sultana et al., 2021 [35] (Canada)	Quasi-experimental RACF	Twenty-four participants aged ≥65 years with documented moderate to severe dementia (Cognitive Performance Scale CPS of three to five) with at least one responsive behaviour within the past 4 weeks. Mean age 85.8 years and 75% (*n* = 18) were female. Mean CPS score was 3.4. 87.5% (*n* = 21) had unspecified dementia type.	Individual sessions of 30 min each, five days a week for two weeks. VR via a projector that provides 3D visual and auditory experiences without a headset. A customised multimedia library of 360-degree video scenes and music was produced based on family member’s responses. Examples include farm, cherry blossom, truck driving, or dolphin swim club. Same customised library item is replayed for participants unless requested otherwise.	Data were collected before and two weeks after the intervention. CSDD and CMAI were used to assess depression and agitation.	Mean VR session was 22.2 min. At least 18 participants (75%) were able to complete 80% of the planned sessions.	No adverse events observed.	No difference in depression pre and post-VR (Effect size (ES) = 0.4; ES considered clinically meaningful when >0.2). No difference in agitation pre and post-VR (ES = 0.2). Dose of prescribed antipsychotic drugs were reduced for 33% (*n* = 8) participants after intervention ended.

### Data synthesis and presentation

Findings were presented through a narrative synthesis. Meta-analyses were conducted using JBI SUMARI [36] on studies that had accessible data and reported comparable outcomes. Study authors were contacted via email to request relevant missing data; however, some responses were not received in time for publication. The inverse variance method using a fixed-effects model was applied to calculate the mean difference (MD) and the standardised mean difference (SMD), since the same outcomes were assessed using different tools in the included studies [37]. A fixed-effects model was selected due to the limited number of studies in each analysis [38]. Statistical heterogeneity was evaluated using chi-squared and I-squared tests.

## Results

### Selected studies

The database search yielded 112 papers, including 37 duplicates. Review of the titles and abstracts of the remaining 75 papers identified that 53 did not meet the inclusion criteria and so were removed. This left 22 papers that were retrieved and reviewed in full-text. After screening these papers, 10 studies were found to meet the inclusion criteria. See Appendix 2 in the Supplementary Data section for PRISMA diagram of study selection.

#### Study characteristics

The ten included studies consisted of one RCT [26] and nine quasi-experimental studies [20, 27–32, 34, 35] (Table 1).

#### Setting

Studies were conducted across seven countries, including Canada (*n* = 3, 30%), Australia (*n* = 2, 20%), Cyprus (*n* = 1, 10%), Portugal (*n* = 1, 10%), Spain (*n* = 1, 10%), Taiwan (*n* = 1, 10%) and the United Kingdom (*n* = 1, 10%). Two (20%) studies were conducted in a hospital setting with acute inpatients in a medical ward [20, 26]. Six studies (60%) were undertaken in an out-of-hospital care facility, with three (30%) in long-term residential aged care facilities (RACF) [27, 32, 35], two (20%) in dementia care units [28, 30] and one (10%) in a dementia-specific RACF [31]. The remaining two studies (20%) were delivered in the community, one (10%) by a local service provider to older people [29] and the other by a day-stay unit [34].

#### Participants

Studies involved participants with mean ages ranging from 73 to 89 years, except one study where the mean age was not specified [27]. All studies had more than 50% female participants. Only three (30%) studies included participants with dementia diagnoses based on guidelines, including the Aged Care Funding Instrument [27], the National Institute on Aging-Alzheimer’s Association Revised Clinical Criteria for Alzheimer’s Disease [30] and the Minimum Data Set—Cognitive Performance Scale Score [35]. The other seven (70%) studies included people with a clinical or documented diagnosis of dementia. The severity of cognitive impairment and scores used to assess cognitive ability varied between studies; however, most included people with dementia of varying severity, from mild to severe.

#### Usage and dosage of VR

The duration, frequency and length of VR sessions varied between the studies. Nine (90%) studies used individual VR sessions, whereas one (10%) performed group VR sessions [27]. The mean session length varied from 1 min [28] as part of standard occupational therapy sensory sessions to 22 min [35]. The frequency of sessions also varied from one session to a defined frequency of two to five times a week.

Most studies (*n* = 9, 90%) used fully immersive VR characterised by HMD, with only one (10%) study using a projector displaying 360-degree video recordings [35]. The VR experience consisted of a series of short 360-degree films from a pre-selected video pool. The VR scenes showcased natural scenery like forests, lakeshores, and beaches and specific locations such as streets, squares, historical landmarks, or travel destinations. Specific scenes were able to be selected based on participant preference in seven (70%) studies [20, 26–29, 35], with two (20%) studies specifically creating videos based upon information provided by caregivers seeking to elicit positive memories [29, 30].

### Quality assessment

Due to the absence of a clear cut-off for exclusion based on quality criteria, the limited number of included studies and their overall good methodological quality, no papers were excluded based on quality assessment. Seven studies (70%) were rated as high quality (‘yes’ responses to ≥70%) [27–31, 34, 35] and three studies (30%) were of medium quality (‘yes’ responses to 50%–69%) [20, 26, 32] (see Appendix 3 and 4 in the Supplementary Data section for the full details of the quality assessment). Only two studies had a control group [26, 27], limiting the ability to compare intervention effects against standard care or other interventions.

### Review findings

The findings are presented in two sections: (i) the impact of VR on BPSD, and (ii) acceptability, optimal dosage, and safety of VR.

### Impact of VR on BPSD

All ten studies assessed BPSD outcomes in participants. Each study examined different combinations of BPSD symptoms, including overall BPSD scores, aggression, agitation, depression, anxiety and apathy.

#### Overall BPSD scores

**Figure 1:**
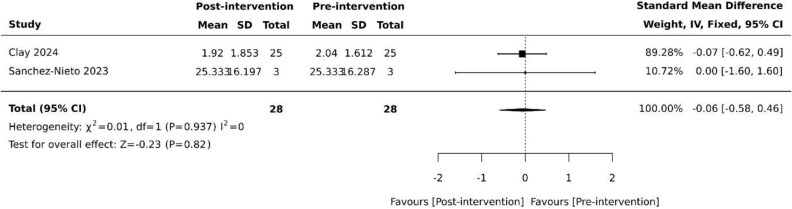
Effect of VR on overall BPSD based on NPI. SD: standard deviation; CI: confidence intervals.

Overall BPSD scores were collected in four (40%) studies [26, 28, 29, 34], using the neuropsychiatric inventory (NPI) as the basis for assessing overall changes in BPSD. However, each study employed slightly differing methodology [26, 28, 29, 34]. The standardised NPI scale was used by Coelho et al. [29], while the NPI-questionnaire (NPI-Q) was used by Sanchez-Nieto et al. [34]. Appel et al. [26] used key terms from daily nursing notes on BPSD symptoms to categorise into three predetermined behaviour clusters (NPI-10-like, violence and wandering) based upon NPI-10 and the difference in mean number of events was analysed. Clay et al. [28] reviewed clinical records retrospectively and recorded neuropsychiatric symptoms using the NPI as its framework. None of the four studies revealed statistically significant differences in overall NPI pre and post-VR intervention [26, 28, 29, 34]. Despite all studies using NPI as the basis for their measurements, additional data needed for standardisation was unavailable. Pooled results from the two studies with available data [28, 34] revealed no effect on overall BPSD (SMD −0.06, 95% −0.58 to 0.46, *P* = .82) with minimal heterogeneity (Figure 1).

#### Aggression

Three studies (30%) reported the impact of VR on aggression [26, 27, 31]. Aggression was evaluated using the Overt Aggression Scale—Modified for Neurorehabilitation (OAS-MNR) [31] and categorising aggressive symptoms into predetermined NPI clusters [26]. Appel et al. [26] found a reduction in physically aggressive behaviours and loud vocalisations in the intervention group versus the control group in an acute inpatient setting (*P* = .01). Similarly, Matsangidou et al. [31] noted reduced frequency and severity of aggression from an aggregate OAS-MNR aggression score of 9 at baseline to 0 during and immediately after VR therapy (no p-value was reported). However, Brimelow et al. [27] found no difference in the frequency of behavioural incidents when analysing the behaviour report logs in their electronic medical records (*P* = .864).

#### Agitation

**Figure 2:**
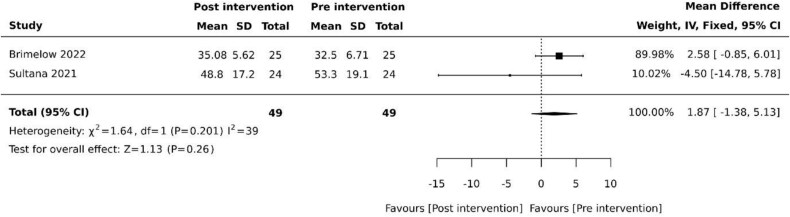
Effect of VR on agitation. SD: standard deviation; CI: confidence intervals.

Agitation was evaluated using the Cohen-Mansfield Agitation Inventory (CMAI) following intervention periods of three weeks and two weeks in two (20%) studies, respectively [27, 35]. Pooled studies revealed no effect on agitation (MD 1.87, 95% −1.38 to 5.13, *P* = .26) with moderate heterogeneity (Figure 2).

#### Depression

**Figure 3:**
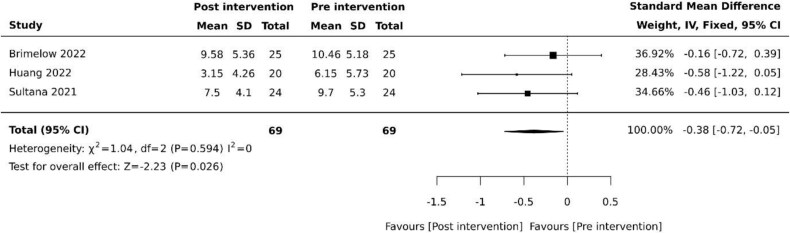
Effect of VR on depression. SD: standard deviation; CI: confidence intervals.

Three (30%) studies examined the effects of VR on depression [27, 30, 35]. Of these studies, two (20%) used the Cornell Scale for Depression in Dementia (CSDD) [27, 35] and one (10%) used the Centre for Epidemiological Studies Depression Scale (CESD) [30]. Pooled results revealed reduced depressive symptoms following VR therapy (SMD −0.38, 95% −0.72 to −0.05, *P* = .026) with minimal heterogeneity (Figure 3).

#### Anxiety

Three (30%) studies assessed the impact of VR on anxiety using different tools: General Anxiety Disorder 7 item (GAD-7) [27], the Hamilton Anxiety Rating Scale (HARS) [34] and the Observed Emotion Rating Scale (OERS) [29]. Brimelow et al. [27] showed no change in anxiety with GAD-7 scores of 4.27 before and 4.06 after the 3-week intervention period (*P* = .681). GAD-7 is typically used for screening with cut-off scores of 10 for generalised anxiety disorder, which indicates no anxiety at baseline in this study. Similarly, Sanchez-Nieto et al. [34] showed no difference in both the psychological and somatic anxiety items using the Hamilton Anxiety Rating Scale (HARS) between baseline and week five of the study after three weeks of VR intervention (*P* > .05). However, Matsangidou et al. [31] demonstrated reduced anxiety symptoms using OERS, from a rating of 2.65 before to 1.2 immediately after VR exposure based on a dementia-specific RACF (*P* < .001).

#### Apathy

Two (20%) studies examined the effects of VR on apathy [27, 32]. Both studies assessed apathy using the Person Environmental Apathy rating (PEAR) scale [27, 32]. The multiple-session group VR programme conducted by Brimelow et al. [27] demonstrated a reduction in apathy immediately after each VR session (*P* < .001). Meanwhile, Moyle et al. [32] demonstrated no change in apathy with a PEAR apathy score of 18.3 before and 18.7 after the singular VR session (*P* > .05). However, there was reduced apathy during the VR session compared to baseline (*P* = .01) [32].

### Acceptability, optimal dosage and safety of VR

Acceptability was reported in nine (90%) studies [20, 26–29, 31, 32, 34, 35] and safety outcomes were reported in six (60%) studies [20, 26–29, 35]. Acceptability was reported by either the percentage of participants who completed their proposed session numbers [26–29, 34, 35] or intended VR exposure duration [31], recording any participant requests for more sessions or longer sessions [20, 31] or the mean duration of VR sessions completed [20, 26, 31, 32, 35]. The intended total of four and nine VR sessions was completed by 78% [29] and 100% [34] of participants in each study, respectively. 85% of participants completed the full intended VR exposure duration of 15 min [31]. 70% of participants opted for extra sessions during their hospital stay [20], while 85% of participants requested longer exposure times than the planned 15 min [31]. The actual mean duration of VR sessions tolerated by patients ranged from 6.2 to 22 min in the five (50%) studies that reported this outcome [20, 26, 31, 32, 35].

Adverse events related to VR intervention were uncommon and mild, with two cases of eye strain and head fullness [29], two cases of headache and giddiness [27] and two cases of mild nausea [20, 26]. The headset was considered too heavy in one participant [20] and caused discomfort due to ill fit in two participants [27].

## Discussion

This systematic review has examined the effectiveness of VR as a non-pharmacological therapy for reducing the severity of BPSD and explored its acceptability, safety and optimal dosage. Despite mixed results, several studies identified potential positive benefits, including reduced aggression [26, 31], depressive symptoms [27, 30] and apathy [27, 32]. Favourable acceptability and minimal side effects were observed across various clinical settings, supporting VR as a potential intervention for reducing BPSD severity.

The impact of VR on overall BPSD yielded mixed findings. Several studies failed to demonstrate statistically significant changes using NPI-based tools. While NPI is a validated clinical tool for dementia-related psychopathology, it is typically used to define behavioural changes over a 4-week period [39]. All but one of the included studies had shorter assessment periods, which may explain the limited statistically significant results. Additionally, BPSD symptoms are complex and dynamic, often relying on proxy reporting [40], which further complicates assessment with standardised tools. The NPI may not have fully captured the nuanced effects of VR on BPSD in the short term, highlighting the need for more sensitive and specific tools to measure BPSD.

Aggression and agitation are among the most challenging BPSD symptoms [41], often treated with antipsychotic medications despite the increased risk of death, cerebrovascular adverse events and extrapyramidal side effects of pharmacotherapy [42]. Triggers such as pain, noise, and environmental stimulation in hospitals may exacerbate these behaviours [43]. More evidence-based non-pharmacological interventions for BPSD are needed. This review found that VR reduced aggression in both an acute hospital and dementia-specific RACF setting [26, 31]. VR-based relaxation devices have also alleviated discomfort, pain, stress and anxiety in ICU patients [44], while VR calm rooms have successfully distracted psychiatric patients from their stressful extended hospital stays [45]. These findings highlight VR as a potentially safe alternative to antipsychotic medications to reduce aggression and agitation that warrants further research.

Anxiety and depression are among the earliest non-cognitive expressions of BPSD [46]. This review found that VR could be effective in reducing depressive symptoms [27, 30], mirroring findings from Zhai et al. [47] where VR reduced depression in RACF residents. This is relevant given the higher rates of depression and anxiety associated with moving into RACF [48]. VR may help ease this transition by providing new experiences and social interactions. This review showed mixed results in terms of anxiety reduction. Interestingly, there has been growing research on VR reducing perioperative anxiety by easing anticipatory fear [49]. During the COVID-19 pandemic, where those in COVID-19 intensive care units faced prolonged isolation periods, VR reduced perceived stress and anxiety in those with mild cognitive impairment [50]. This may translate to similar benefits in older people with dementia who face social isolation in hospital or institutional settings. Future studies are necessary to confirm VR’s effects on mood and emotions of older people with dementia.

This review demonstrated that people with dementia tolerated VR well across hospital, RACF and community settings, with few adverse events. Completion rates were high, even among acutely unwell medical inpatients, with comparable adherence (65%–78%) to exercise programmes for those with BPSD [51]. Recorded adverse events in this review were much lower than the side effects of nausea or headache for common antidepressants [52]. Symptoms such as eye strain, headache and mild dizziness were likely caused by motion sickness or headset weight [53]. Much like music therapy for BPSD, which can cause overstimulation with excess volume [54], VR experiences should be carefully adjusted for comfort, particularly in those with pre-existing motion sickness or visual impairment. Encouragingly, a systematic review by Chen et al [55] found no negative outcomes with VR exergames in older people, despite their demands on movement and balance. Saredakis et al. [56] also reported lower levels of cybersickness in older participants. There is a need for further research to refine candidate selection and enhance VR comfort and design for broader use.

As has been seen in reviews of interventions in other disease groups [57], the included studies used widely different VR protocols. Currently, there is limited guidance on dosage of VR and its impact on outcomes as no studies assessed how these variations in protocol impacted the effectiveness in reducing BPSD severity. This variability, together with the lack of standardised BPSD outcomes, complicates comparisons and determination for the ideal VR dosage. A recent meta-analysis found that the most effective VR-based rehabilitation for cognition and motor function in individuals with mild cognitive impairment or dementia was 30 min, three times per week for five to eight weeks [58]. However, this review [58] focused on community settings, which leaves a gap for hospital-based studies that may also introduce additional variables like logistical constraints and clinical events that could affect adherence [59]. Additional research is needed to compare different VR dosages within the same clinical setting to establish the most effective approach.

This systematic review was the first to explore the use and efficacy of VR on BPSD. It consolidates current knowledge on how it is used, its potential impact on specific BPSD symptoms as well its acceptability and safety. The review also highlights the heterogeneity in the literature and provides valuable insights to guide future research. However, several limitations must be acknowledged. The strength of the evidence is limited by the small number of studies undertaken across limited geographical regions, additionally, issues such as small sample sizes and lack of blinding impact study quality. Nine out of ten studies were observational with no control group nor blinding, increasing the risk of bias and limiting the ability to draw conclusions. The heterogeneity of interventions, including variations in VR equipment, frequency, duration, imagery, and interventions settings, as well as differences in dementia type and severity, further compromise the generalisability of findings. Studies were short (up to three months) with limited follow-up, limiting assessment of longer-term sustained effects. Methodological limitations must be also considered. The search strategy, while comprehensive, may have excluded relevant studies due to language restrictions, as only English-language publications were included. Additionally, variations in search terms and indexing of VR-related interventions may have led to the omission of some studies. A meta-analysis could not be completed due tothe variability in study designs, outcome measures, and missing data. Where meta-analysis was performed, the absence of control groups meant that only pre-post comparisons were possible, making the results highly susceptible to bias. Although this review represents the best available evidence, given these limitations, findings should be interpreted with caution.

## Conclusion

While VR may become a non-pharmacological intervention for reducing the severity of BPSD, the current evidence is insufficient to establish its effectiveness definitively. Nonetheless, VR has demonstrated an excellent acceptability and safety profile across numerous settings. The limited number of studies, varied methodology, and quality limitations constrain the findings, underscoring the need for future research with larger sample sizes, standardised VR intervention protocols and comprehensive BPSD outcome measures. Integration of VR into clinical practice could potentially alleviate the challenges faced by both patients and caregivers.

## Supplementary Material

aa-24-2585-File002_afaf117
