# Supplementary material for: The use and effect of virtual reality as a non-pharmacological intervention for behavioural and psychological symptoms of dementia: a systematic review and meta-analysis
Source: Age Ageing. 2025 May 12;54(5):afaf117. doi: 10.1093/ageing/afaf117 (PMC12068490; doi:10.1093/ageing/afaf117)
Supplement: aa-24-2585-File002_afaf117 [file aa-24-2585-file002_afaf117.docx]

**The use and effect of virtual reality as a non-pharmacological intervention for behavioural and psychological symptoms of dementia (BPSD): a systematic review and meta-analysis**

**APPENDIX 1**

**Search Strategy**

Table A1 – PUBMED search on 12 March 2024

| **Search number** | **Query** |
| --- | --- |
| #1 | Virtual reality OR VR OR virtual reality[mh] |
| #2 | dementia[mh] OR Alzheimer disease[mh] OR dementia[title/abstract] OR alzheimer*[title/abstract] OR vascular dementia [title/abstract] OR lewy body dementia[title/abstract] OR frontotemporal dementia[title/abstract] |
| #3 | behavioral and psychological symptoms of dementia[title/abstract] OR BPSD[title/abstract] OR challenging behav*[title/abstract] OR disturbing behav*[title/abstract] OR difficult behav*[title/abstract] OR disruptive behav*[title/abstract] OR behav* concern[title/abstract] OR agitat*[title/abstract] OR restless*[title/abstract] OR apathy[title/abstract] OR social* disinhibit*[title/abstract] OR sexual* disinhibit* [title/abstract] OR neuropsychiatr* [title/abstract] |
| #4 | 1 and 2 and 3 |
| #5 | #4 filter: English language, 2014-2024 |

Table A2 – EMBASE search on 12 March 2024

| **Search number** | **Query** |
| --- | --- |
| #1 | (virtual reality or VR).mp or exp virtual reality/ |
| #2 | (dementia or Alzheimer* or vascular dementia or frontotemporal dementia or lewy body dementia).mp or exp dementia/ |
| #3 | (BPSD or "behavioral and psychological symptoms of dementia" or challenging behav* or disturbing behav* or difficult behav* or disruptive behav* or behav* concern or agitat* or restless* or apathy or social* disinhib* or sexual disinhib* or neuropsychiatr*).ab,kw,ti. |
| #4 | 1 and 2 and 3 |
| #5 | Limit 4 to (English language and year “2014-current” |

Table A3 CINHAL search on 12 March 2024

| **Search number** | **Query** |
| --- | --- |
| #1 | MH “dementia+” OR MM “Alzheimer’s disease” OR TI (dementia OR Alzheimer* OR vascular dementia OR frontotemporal dementia OR lewy body dementia) OR AB (dementia OR Alzheimer* OR vascular dementia OR frontotemporal dementia OR lewy body dementia) |
| #2 | MH “virtual reality” OR TI (VR OR virtual reality) OR AB (VR OR virtual reality) |
| #3 | AB (BPSD or "behavioral and psychological symptoms of dementia" or challenging behav* or disturbing behav* or difficult behav* or disruptive behav* or behav* concern or agitat* or restless* or apathy or social* disinhib* or sexual disinhib* or neuropsychiatr*) OR TI (BPSD or "behavioral and psychological symptoms of dementia" or challenging behav* or disturbing behav* or difficult behav* or disruptive behav* or behav* concern or agitat* or restless* or apathy or social* disinhib* or sexual disinhib* or neuropsychiatr*) |
| #4 | 1 AND 2 AND 3 |
| #5 | Limit 4 to (English language and year “2014-current” |

Table A4 SCOPUS search on 12 March 2024

| #1 | TITLE-ABS-KEY (“virtual reality” OR “VR) |
| --- | --- |
| #2 | TITLE-ABS-KEY (“dementia” OR “Alzheimer* OR vascular dementia OR frontotemporal dementia OR lewy body dementia) |
| #3 | TITLE-ABS-KEY (“behavioral and psychological symptoms of dementia” OR “BPSD” OR “challenging behav*” OR “disturbing behav*” OR “difficult behav*” OR “disruptive behav*” OR “behav* concern” OR “agitat*” OR “restles*” OR “apathy” OR “social disinhib*” OR “sexual disinhib*” OR “neuropsychitr*” |
| #4 | #1 AND #2 AND #3 |

**APPENDIX 2**

**
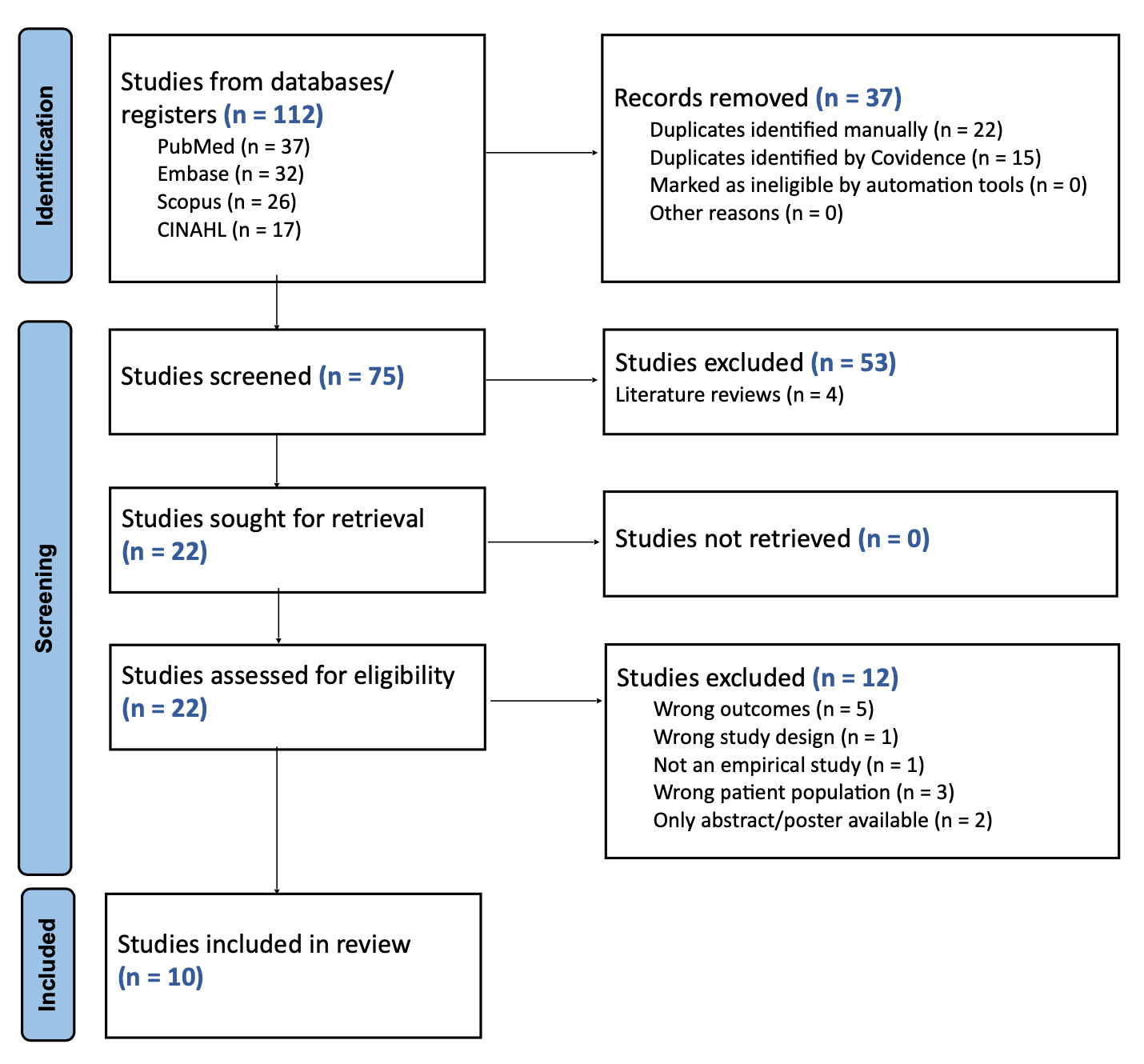
**

Figure: PRISMA Diagram of study selection

**APPENDIX 3**

Table Appendix 3 JBI critical appraisal checklist: RCTs

| **Study** | **Q1** | **Q2** | **Q3** | **Q4** | **Q5** | **Q6** | **Q7** | **Q8** | **Q9** | **Q10** | **Q11** | **Q12** | **Q13** | **% Yes** | **Risk** |
| --- | --- | --- | --- | --- | --- | --- | --- | --- | --- | --- | --- | --- | --- | --- | --- |
| Appel et al., 2024 [29] | Y | N | Y | N | N | Y | N | Y | Y | Y | Y | Y | Y | 69% | Mod |

Q1. Was true randomization used for assignment of participants to treatment groups? Q2. Was allocation to treatment groups concealed? Q3. Were treatment groups similar at the baseline? Q4. Were participants blind to treatment assignment? Q5. Were those delivering treatment blind to treatment assignment? Q6. Were outcomes assessors blind to treatment assignment? Q7. Were treatment groups treated identically other than the intervention of interest? Q8. Was follow up complete and if not, were differences between groups in terms of their follow up adequately described and analysed? Q9. Were participants analysed in the groups to which they were randomised? Q10. Were outcomes measured in the same way for treatment groups? Q11. Were outcomes measured in a reliable way? Q12. Was appropriate statistical analysis used? Q13. Was the trial design appropriate, and any deviations from the standard RCT design (individual randomization, parallel groups) accounted for in the conduct and analysis of the trial? N = No; U = Unclear; Y = Yes. >70% = low risk of bias; 50%–69% = moderate risk of bias; <50% = high risk of bias.

**APPENDIX 4**

Table Appendix 4 JBI critical appraisal checklist results: quasi-experimental studies

| **Study** | **Q1** | **Q2** | **Q3** | **Q4** | **Q5** | **Q6** | **Q7** | **Q8** | **Q9** | **%**  **Yes** | **Risk** |
| --- | --- | --- | --- | --- | --- | --- | --- | --- | --- | --- | --- |
| Appel et al., 2021 [20] | Y | Y | Y | N | N | Y | N | N | Y | 56% | Mod. |
| Brimelow et al., 2022 [30] | Y | Y | Y | Y | Y | Y | Y | Y | Y | 100% | Low |
| Clay et al., 2023 [31] | Y | Y | Y | N | N | Y | Y | Y | Y | 78% | Low |
| Coelho et al., 2020 [30] | Y | Y | Y | N | Y | Y | Y | Y | Y | 89% | Low |
| Huang et al., 2022 [33] | Y | Y | Y | N | Y | Y | Y | Y | Y | 89% | Low |
| Matsangidou et al., 2023 [34] | Y | Y | Y | N | Y | Y | Y | Y | Y | 89% | Low |
| Moyle et al., 2018 [35] | Y | Y | N | N | U | Y | Y | Y | Y | 67% | Mod. |
| Sanchez-Nieto et al., 2023 [36] | Y | Y | Y | N | Y | Y | Y | Y | Y | 89% | Low |
| Sultana et al., 2021 [37] | Y | Y | Y | N | Y | Y | Y | Y | Y | 89% | Low |

Q1. Is it clear in the study what is the “cause” and what is the “effect” (i.e. there is no confusion about which variable comes first)? Q2. Were the participants included in any comparisons similar? Q3. Were the participants included in any comparisons receiving similar treatment/care, other than the exposure of the intervention of interest? Q4. Was there a control group? Q5. Were there multiple measurements of the outcome both pre and post the intervention/exposure? Q6. Was follow up complete and if not, were differences between groups in terms of their follow up adequately described and analysed? Q7. Were the outcomes of participants included in any comparisons measured in the same way? Q8. Were outcomes measured in a reliable way? Q9. Was appropriate statistics analysis used? N = No; U = Unclear; Y = Yes. >70% = low risk of bias; 50%–69% = moderate risk of bias; <50% = high risk of bias.
